# Patients’ body image after open abdominal surgery of abdominal aortic aneurysm – Perceptions and experiences

**DOI:** 10.1002/nop2.1225

**Published:** 2022-04-28

**Authors:** Monica Pettersson, Erney Mattsson, Ingegerd Bergbom

**Affiliations:** ^1^ Sahlgrenska Academy Institute of Health and Care Sciences University of Gothenburg Gothenburg Sweden; ^2^ Deptartment of Hybride and Interventional procedures Vascular Unit Sahlgrenska University Hospital Gothenburg Sweden; ^3^ Department of Vascular Surgery St Olavs Hospital Norwegian University of Science and Technology Trondheim Norway

**Keywords:** abdominal aortic aneurysm, body image, caring science, experiences, hermeneutics, interviews, nursing, open surgery, qualitative

## Abstract

**Aims:**

To explore and gain an understanding of patients’ perceptions and experiences of their body and bodily function in connection with open surgery of abdominal aortic aneurysm.

**Background:**

After the operation of an abdominal aortic aneurysm (AAA) it may be difficult for the patients to understand what the procedure means.

**Design:**

The design was descriptive and based on an analysis of 13 audio‐taped consultations with patients undergoing open surgery for AAA at a Swedish university hospital. The patients’ age varied from 57–79 and the mean age was 71 (70.5 female).

**Methods:**

A hermeneutic approach was used whereby patients were interviewed and draw a picture of their thoughts and experiences of the surgery and what had been done in their bodies. Once patients had finished their drawings, the interviewer asked what thoughts and experiences they had of the AAA.

**Results:**

Patients described experiences as a process of “going from broken to whole again.”

## 1 INTRODUCTION

Not having any symptoms and not sensing that something inside the body is “wrong” can lead to a person feeling uncertain about their ability to perceive their body. Only 11% of patients who operated for AAA had symptoms of their disease prior to surgery (Swedvasc, 2011). Having control of the body and its functions is essential for the human being (Van Manen, 1998). Through the body, humans have access to their senses and can feel changes in connection with illness, suffering and care (Furnham et al., 2002; Lindwall, 2004). According to Gadamer (1996, p. 78), the life of the body is experienced as a constant movement between the loss of equilibrium and a search for a new point of stability. Moreover, illness as a disturbance makes us aware of our bodily nature. Health can be understood as a condition of equilibrium, but the nature of health is hidden, and disease can exist without symptoms. However, it is therefore important for nurses and doctors to understand how patients comprehend the implications of their AAA surgeries and how they perceive their changed bodies due to the surgical intervention and the disease.

## 2 BACKGROUND

Abdominal aortic aneurysm (AAA) is a serious diagnosis and condition, which is often treated surgically. AAA can lead to a life‐threatening rupture, 5.5 cm is deemed to be the size at which the benefits of repair outweigh the risks (Lederle et al., 2002). The total mortality with rupture is about 81%, which is 1%–2% of all deaths in men over the age of 65 (Reimerink et al., 2014). AAA can be operated with two different methods: open repair (OR) or endovascular aortic repair (EVAR). OR entails replacing the aneurysm with an artificial graft, while EVAR is a less invasive procedure that involves introducing a stent graft into the aorta via the arteries in the groin. The number of EVAR procedures has increased and in 2019, the figures for EVAR were 493 and for OR 288 (Swedvasc, 2020). The choice of treatment methods is influenced by the risk of complications, the patient's vascular anatomy, age and other diseases.

In most cases, treatment with EVAR is a comparatively minor physical and physiological insult from which most patients recover rapidly (Pettersson & Bergbom, 2010). It was found that patients who underwent EVAR felt that the surgery was easier than expected, and that it was difficult to comprehend that their bodies had undergone surgery for a serious diagnosis when they only had two small incisions in the groin. The EVAR method usually requires no intensive care and hospital stay duration is shorter (Malina et al., 2000).

Many patients undergoing surgery cannot understand or explain what has been done in their bodies or how these surgical measures can affect them (Pettersson & Bergbom, 2010). To make it understandable patients construct an image in their minds. Such an image can be presented in a drawing and explained or discussed with the caregiver or interviewer. Drawings and conversations/dialogue have previously been used in studies concerning body images and experiences (Bergbom et al., 2017; Modh et al., 2011). A picture could also make it easier to establish contact between the participants (interviewees) and the interviewer.

We claim that it is important that patients are informed about and understand what the surgical procedure means and consists of, that is, what has been done in their bodies, so that they can feel safe both pre‐ and postoperatively. It is therefore vital to investigate and gain knowledge of the patients’ own experiences of their bodies and bodily function in connection with operations for open AAA and the image they create of the intervention. Such knowledge creates an opportunity to improve the pre‐ and postoperative information for the patient undergoing surgery for AAA.

### 2.1 Theoretical frame

In this study, patients’ imagination/perception and experience of their body outcome of the surgical procedure are described/presented using drawings and patients’ verbal expressions. In the drawings and in the participants’ expressions and explanations of their drawings, their experiences and thoughts, that is, the lifeworld is presented. According to Merleau‐Ponty (1997), the lifeworld is the world as the person experiences it including how we also experience our body and he emphasized the body as the primary site of knowing the world. When we interpret pictures, we do so on the basis of our pre‐conceptions and historicity, which is why it is easy to quickly work out what a picture represents and means. In semiotics, this is called the denotation of the picture (Sonesson, 1989). Gadamer (1989) asserts that a picture has its own “being” – it presents itself and has its own reality where the “thing,” that is, what is in focus that is what has been changed in the body, is represented. This enables healthcare staff to tailor information to the patient's needs so that they feel informed about their disease and participate in their treatment. The patient's understanding of what the surgical intervention means concerning changes in the patient's body could be seen as a valid consent for surgery.

Fantasies and notions about what has been done inside the body in connection with surgical procedures can lead to anxiety and this can affect body image (Lindwall & Bergbom, 2009). Price (1990) uses three aspects to describe normal body image: the body ideal, which is a mental image of how the body should look, feel and behave, how the body is in reality and how it is presented, meaning how the body is adapted and presented to other people. The disease affects our relationship with our bodies and with ourselves and life itself. The disease can also mean that we have to live with a changed body. By encouraging people to talk about and illustrate their experiences and ideas about what an AAA intervention or surgery has done in their bodies, we are able to gain an understanding of patients’ body image. Such knowledge of patients’ experiences and the perception they have of the intervention is essential for developing care in discussions with the patient both before and after the operation.

## 3 THE STUDY

### 3.1 Aim

The aim was to explore and gain an understanding of patients’ perceptions and experiences of their body and bodily function in connection with open surgery of AAA. Questions**:** What image do the patients have of how the aortic aneurysm and surgical interventions look inside the body? What experiences of the body and bodily function after diagnosis and the surgical procedure do patients report?

### 3.2 Design

The design of this study was descriptive and explorative and based on an analysis of transcriptions of audio‐taped interviews with patients who described their drawings and experiences after undergoing OR for AAA at a Swedish university hospital. Drawings as a method for describing chronic pain have been used by Phillips et al. (2015) using Rose’s (2007) visual methodology. Guillemin (2004) used drawings when exploring people's experiences and understanding of illness. A qualitative method with a hermeneutic approach was used, as the aim was to explore and gain a deeper understanding of patients’ perceptions, images and experiences of their body after surgery. In Gadamer's hermeneutic philosophy (1989) texts, plays and pictures or art can be interpreted and understood.

### 3.3 Study participants

Patients were recruited in 2017 for the study from a vascular surgery unit at a university hospital in Sweden. The patients included in the study were only those that had open surgery as the alternative, thereby excluding those patients where an endovascular repair was excluded according to locally defined criteria. The inclusion criteria were should undergo elective OR surgery for AAA due to cardiovascular disease, 18 years and older, able to speak and understand Swedish. The first author invited twelve patients consecutively to participate in the study when they were admitted to the vascular surgery unit before surgery. In connection with this invitation, patients received oral and written information about the study. All the invited patients agreed to participate (nine men and three women). The patients’ age varied from 57–79 and the mean age was 71 (70.5 female). Five patients were married and only one patient was still working.

### 3.4 Data collection

**FIGURE 1:**
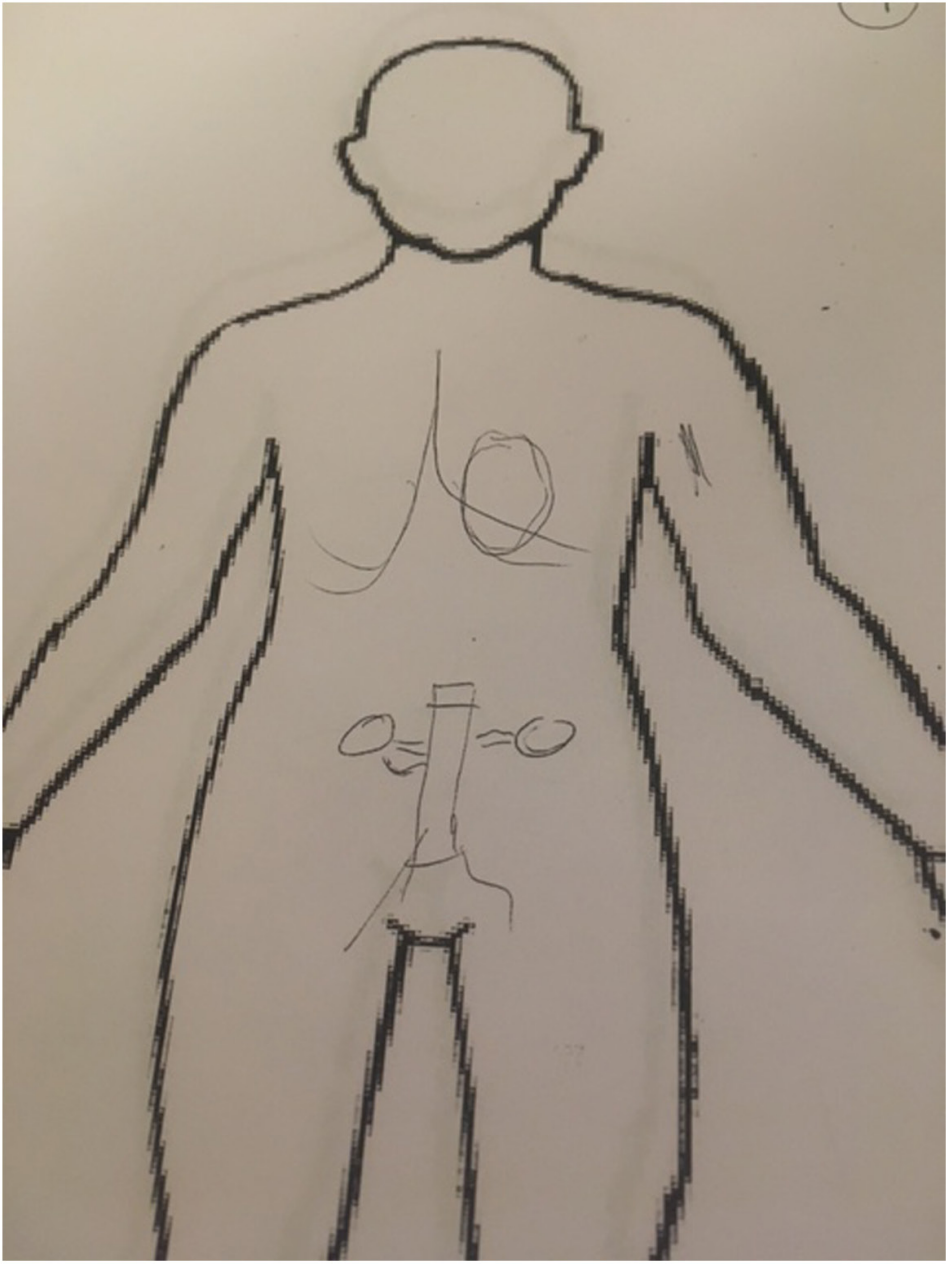
Patient picture of how they imagined the surgical intervention, example 1

**FIGURE 2:**
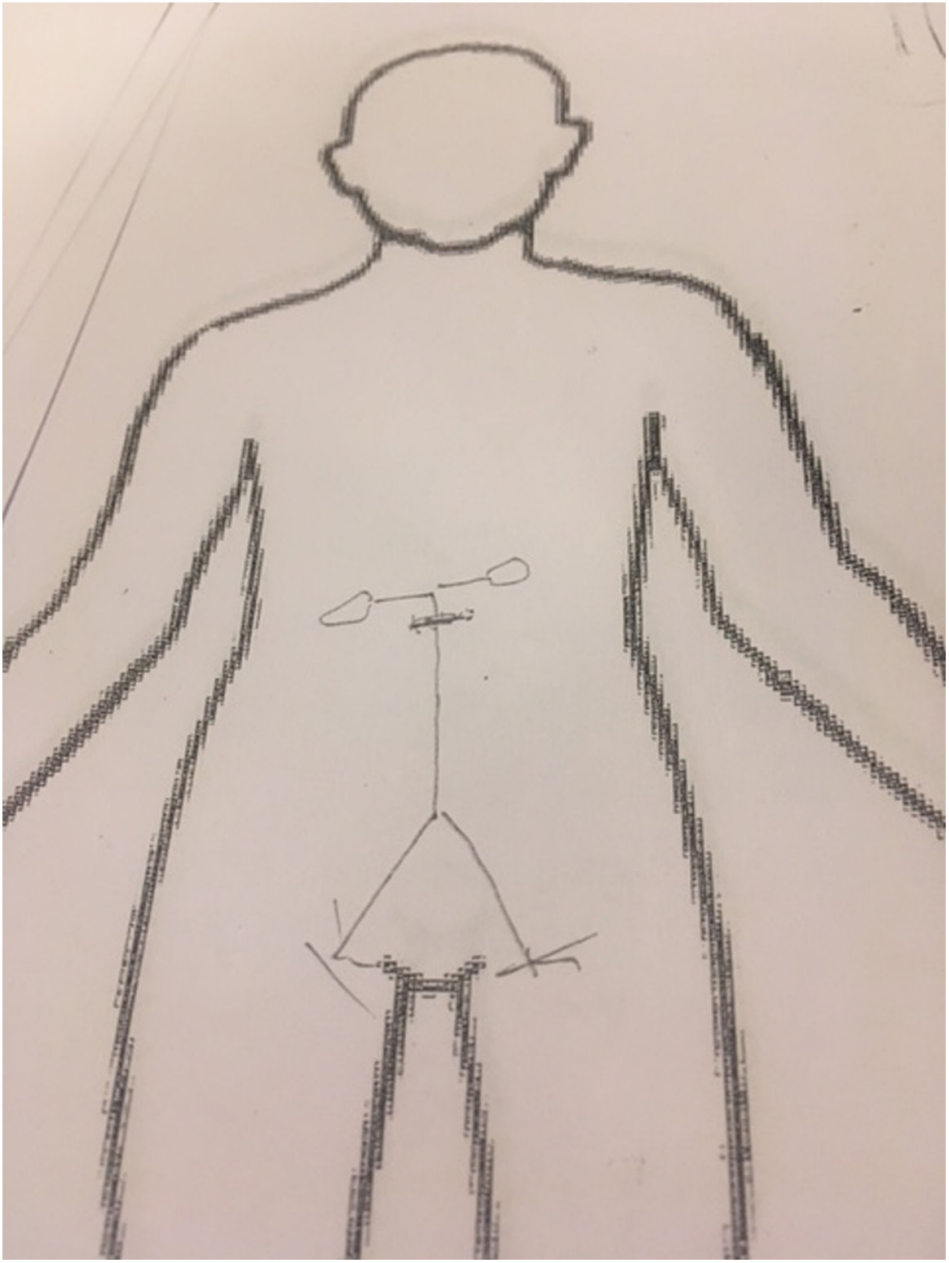
Patient picture of how they imagined the surgical intervention, example 2

All patients were interviewed 1 month after surgery, by the first author, when they returned to the vascular outpatient clinic at the hospital. The interview started by asking the patient to draw a picture of what they imagined the surgical intervention meant and what they thought had been changed and how it looks inside their bodies, Figures 1 and 2. The reason for asking the patients to draw a picture was that, according to Högberg (1996), artwork can contribute to self‐reflection, thus adding depth to the dialogue and making the experience of surgery more concrete. It was the bodily experiences that were of interest and the patients’ imaginations of what the surgery brought, not only feelings and thoughts. By asking about the drawn picture, further explanations were obtained and misunderstandings avoided. By discussing the picture, the researcher's preunderstanding of patients’ awareness and perception of the diagnosis and surgery was challenged. In the period of the study, no illustrations were given from the treating unit to the patients prior to the treatment.

After the patients had finished their drawings, the interviewer asked the patients to describe and explain what they had drawn in relation to how they imagined the body and the results of the surgery. Questions were then asked, such as: How do you imagine your body looks inside after the surgery? What has the surgeon done? How does your body work now? Has your body's function changed after the intervention? What has changed? What are your thoughts about the consequences (risks and side effects) of the surgery? What fears and prospects does the patient have? Do they have existential questions and what are they in that case? The interviewer also asked follow‐up questions. For example, Can you explain more about this? What do you mean? What does this mean? The interviews were audio‐taped and transcribed verbatim. The transcribed text consisted of 104 single‐spaced pages.

### 3.5 Data analysis

The method of Fleming et al. (2003) was used in the process of analyzing the text from the interviews and descriptions of the drawings. The interpretations and understanding that were gleaned are based on the analysis of the interview text, including the conversation concerning the drawing. This analysis consisted of four steps. In the first step, the twelve interviews were read in their entirety to obtain an overview and initial understanding of the text. The aim was to find “otherness” and perhaps something “new” in relation to the researchers’ pre‐understanding. In the second step, the text was read again to identify the text's meaning units, main theme and subthemes. How did the patients explain the experiences and descriptions of their bodies in the drawings and what did it mean for the patients? Were metaphors used? In the third step, the themes and text were read again and interpreted. This involved moving back and forth between the meaning units, themes and subthemes. Finally, in the fourth step, further understanding and reflection were achieved based on the interpretations and understanding that had been gained in the third step. All subthemes were illustrated by one or more quotations (with an example) from the interviews; according to Thorn (2020) such exemplification can strengthen the validity. A similar method of reading and interpreting text based on hermeneutics and where drawings were used has previously been presented by Bergbom et al. (2017) and Bergbom and Lepp (2021).

### 3.6 Validity and reliability

The twelve patients’ drawings were interpreted by a professor emerita in nursing (IB). The interpretation of what the drawings mediated resulted in a text including eight pages. This text was then interpreted by the first author, who identified two themes using Fleming et al's method (2003) for analysis. This researcher had no access to the patient interview text so this person did not know how the patients had explained their drawings. The reason for this “blind” interpretation was that it enabled the patients’ statements and the researchers’ interpretations to be validated. This interpretation of the drawings is presented in the result section as the researcher's interpretation.

### 3.7 Ethical considerations

The Regional Ethical Review Board (diary no. 560–14) approved the project. Patients gave their informed consent prior to participation. The data are kept in a secure location and handled in accordance with the recommendations of the World Medical Association.

## 4 RESULTS

The results are presented in three steps: 1. The researcher's descriptions and interpretation of the drawings. 2. Descriptions and interpretation of the interview text about patients’ perceptions of the surgical intervention in their body. 3. Interpretation and understanding.

### 4.1 Descriptions and interpretation of the drawings (Step 1)

In the researcher's interpretation of the drawings, two themes were identified (appeared): Connection to the heart and life and something isolated from the rest of the body.

#### 4.1.1 Connection to the heart and life

The operation is seen as something being done in the middle of the body and as a relatively major procedure. The heart was seen as having an important function in relation to the operation and can be seen as a metaphor for the supply of blood and for the living heart. The heart was also drawn as a cross, where the intervention stems from, which was interpreted as the patient wanting to convey through the drawing that the intervention was related to the heart and that the heart was involved. There was also an illustration of blood coming from the heart and the heart coloured red. However, in some drawings, it was interpreted that there were no connections between the heart and the surgical intervention or the function of the aorta and its importance for life.

#### 4.1.2 Something isolated from the rest of the body

The graft was illustrated as a tube that was inserted in the body and could be seen as an open flow to the rest of the body, but in most pictures it appears to have been perceived as something quite isolated from the rest of the body. This can be interpreted as a “spare part or repair of individual part” and not linked to the function and the vital context of the rest of the body. The graft in the pictures was interpreted as having the aorta repaired and that it was straight and functional again. In some drawings, there is a bulge on the aorta, and this can be interpreted as a risk of new or persistent bulges or that the blood can collect in these bulges, implying a risk that the body is not fully “repaired.”

### 4.2 Descriptions and interpretation of the interview text (step 2)

In the interpretation and analysis of the interview text, including patient explanation/descriptions of the drawings about patients’ perceptions of the surgical intervention in their body, four themes were identified. For all themes and subthemes, see Table 1.

**TABLE 1:** Themes and subthemes from the interview text

Theme	Subtheme
Getting a disease in the body	Feeling afflicted by disease and realizing how serious it is Getting repaired but unsure of how Entrusting yourself and your body
Handing over control	Experiencing powerlessness Entrusting yourself and your body to the healthcare professionals
The body will be whole again	Being restored Allowing the body to recover
Having a functioning body again	Getting your body back and becoming one with it Leaving something behind you

#### 4.2.1 Getting a disease in the body

##### Feeling afflicted by disease and realizing how serious it is

The participants described it as having something growing inside their bodies and this could be life‐threatening if the operation was not done – they felt afflicted and disappointed by their bodies. Obtaining information about the state of their bodies could lead to a crisis and the informants admitted fear upon learning that their bodies were affected and no longer complete. They described the discovery as if the body did not warn them or produce any symptoms despite not being “whole” and that they felt vulnerable and scared. The patients explained their pictures as “something vital in the body that is broken.” The broken body elicited existential questions about life possibly and suddenly being over. Other patients said they got defensive, thinking that what had happened to them and the upcoming operation did not concern them. The informants said that even their relatives were worried about their condition, and in many cases, were more worried than the patients themselves.

Because all of a sudden I saw myself coming home and sitting down in my favourite chair and having a think about things – Listen Olsson, this'll pop tomorrow and then you'll die in a couple of minutes. If that turns out to be true I don't know – but that's how I was thinking.

I knew that if it breaks, that's not great – not for survival. At least not if it breaks in the wrong place, I mean when you're in the wrong place.

It's probably going to be fine but I think they (the relatives) were actually a bit more nervous than I was.

##### Getting repaired but unsure of how

Although the patients received information about their upcoming operation, they sometimes found it difficult to take it in and comprehend how their bodies would be affected by the operation and what would be done inside them. This uncertainty was usually linked to the attitude of focusing on getting through the operation. When the patients explained what they had drawn on the picture, they described it in terms of the aorta getting repaired and becoming whole again but that they were unsure of how and where the graft would be placed in the body but saw it as a “protection.” All but one patient stated that they were not receptive to detailed information about the operation itself because there was a sense of “unreality.” A sense of this not apply to them personally and that they were out of their bodies was also expressed.

You knew then that it's one of these operations that's coming up but there's a lot that might be coming up that you can never know what will happen with anything.

There was the risk and I even got a little booklet or brochure or whatever and it said there too but still there was this thing that it didn't apply to me.

##### Entrusting yourself and your body

In handing over their bodies for the operation, the patients showed great trust in the doctor. A number of patients stated that they had not considered the risks of the operation beforehand, while others said it was a bit difficult but that they still felt a great trust in them. In most cases, the informants were also able to discuss the type of operation and its effect on the body but said they did not want to make the decision, but felt they trusted the doctor to make the right decision. This is interpreted as the informants wanting to hand over their body to the doctor and that the individual was unable to influence their situation. There was security in feeling they could trust the healthcare professionals.

I haven't thought about it that much, actually… I spoke to xxx and he said nothing can change it and what was I to do – I trusted the doctor and I have asked because I don't want to put other people at risk.

It might have been a bit difficult for me and yet I couldn't do anything about it so they have to take over and I trusted them… completely.

I haven't been pushy – I’ve listened to what the doctor has said… he said not to worry and tell them not to worry so I shouldn't – they're the ones who're trained – it was mainly because I wanted a proper answer, that like, it was nothing to worry about.

#### 4.2.2 Handing over control

##### Experiencing powerlessness

The participants felt they were obliged to have the operation if they wanted to live and therefore had to hand over control of their bodies and their lives to “health care” – at least temporarily. This led to feelings of powerlessness. However, the participants did say that they had been told they could choose whether or not to go ahead with the operation and that the risk of the aneurysm bursting was minimal but there was still anxiety over the fact that their lives were in the balance. Not having a choice means not having control over your body and consequently, your life. At the same time, it felt reassuring that the aneurysm had been discovered and that something could be done. When the patients understood that an operation was necessary, they also understood that it meant handing over and entrusting their bodies and their lives to the doctors and the healthcare staff as they were unable to do anything to improve their situation themselves.

It was because it had burst or, well, it wasn't whole, so to speak. But for me it was more that they had to perform an operation so I just had to go with it.

When we discussed the operation he said that… he asked if I wanted an operation and I said that there isn't a choice and I said, I do trust you, I said.

##### Entrusting yourself and your body to the healthcare professionals

After the operation, the patients said a wish to regain control over their bodies by wanting to find out what the intervention looked like inside the body and what had actually been done. At the same time, they wanted assurances that the graft would be safe and remain intact. They had also been given information about how the operation would be done and in the comments to their drawings, were able to describe that blood could now flow through the whole body, a form of life creation to all organs.

The handover (entrusting your body) was necessary for the patient to get back to their usual or “normal” life again and for them to be able to do what they had previously valued doing. At the same time, the patients emphasized that the operation was their own choice and that it felt like having the operation was life‐critical, meaning that the present danger would be eliminated and the aorta repaired. It was evident they wanted control over their bodies but at the same time wanted to hand over that control and to do so with their bodies. After the operation, questions were raised as to what had been done with their bodies and how these interventions or changes would affect their lives.

I thought it would be a bit wearing, so you have to hand over control of your body.

it was like that and somehow I’d had enough before that – I’d thought about it a lot – and that my body and I could then be best friends, so we've done this together. And I was obliged to hand it over because I couldn't do anything about it myself.

#### 4.2.3 The body will be whole again

##### Being restored

The participants described wanting to be “repaired,” hoping their postoperative condition would not otherwise affect their lives and that everything would be as before – they would not have to think about their condition anymore. They described feeling secure in the knowledge the graft was in position and that they would now be fully healthy and “not leak” – the graft would enable them to live as they did before and their bodies would keep working this way for the rest of their lives. The participants also felt secure because the doctor who operated on them told them they did not have to return to the hospital and could now live normally and put their operations behind them. Nevertheless, despite this sense of security, the participants still had doubts about the repairs that had been made to the aorta and whether it would hold and were worried that it might fail again, despite having the operation. This uncertainty and anxiety affected their daily activities, such as physical training or lifting heavy objects.

Yes, I was told that I don't have to think about it anymore at all, for the rest of my life.

When I went for my check‐up, the doctor said everything's working really well, everything looks really good and you don't have to come back here ever again – and then I thought, if he's saying that then everything must be alright.

I’ve started doing exercise now – well, I could have done ages ago but I’ve been a bit scared that something might happen… I don't know what could happen but… I don't know if it can split or something like that… that's kind of how I think.

##### Allowing the body to recover

The participants stated that recovery after the operation was important and that their bodies were continually working on recovery, which could indicate they viewed the body as separate from their own person. They felt they could trust their bodies and understood “it” needed time to heal after the operation. The participants also described their bodies as needing nourishment, which is associated with the existential aspect of the lived life. Nearly all the patients stated that their bodies had been affected in a number of ways by the operation, primarily in terms of severe fatigue that affected their mobility and could even lead to depression. Their condition and the experience of the operation's effect on their bodies was something many of the patients wanted to get out of their minds as it worried them, that is, that they would not be restored to the condition they were in before the operation.

And afterwards, I was like a zombie, yeah, totally, for one and a half weeks. I mean I was completely finished – I had these terrible nightmares. So I really should have spoken to a psychologist but I’ve never spoken to anyone like that.

My body needed to recover – it's like I wasn't surprised about that. I feel my body has been tired and needed a lot of nourishment and it's working hard with this, healing this wound here.

#### 4.2.4 Having a functioning body again

##### Getting your body back and becoming one with it

It was evident the patients viewed their bodies as something that would warn them if something was wrong and that they should listen to their body's signals. The idea that the body would signal if something was wrong became important to them and something they trusted postoperatively, feeling they had in some way “got through it.” They could let go of the anxiety they had experienced before the operation, as the threat inside their bodies was gone. The body was described as an important part of recovery and that it would once again work as normal. Once the body had been repaired and was whole again, this influenced the participants’ lives as a whole, and they could resume or begin activities that had a positive effect on them. The body could be seen as a part of yourself but also as a partner – that recovery was something you did together.

No, you think you've thought about it and it feels like…you think a little existentially…that now I’ve made it.

Yes, well I do actually, I do think that, I mean the anxiety has gone of course.

think it was kind of beautiful that my body and I were doing this together, yes, I have to say, it really has supported me in this and really been good and worked hard.

##### Leaving something behind you

Although the participants’ bodies got stronger after the operation, they still sometimes reflected on whether everything was alright, and could fluctuate from feeling lively to feeling low. Existential thoughts arose about their having gone through it and putting the condition behind them but also about not being grateful enough. The patients also described being finished with it and ready to move on in life now that their bodies were repaired and that they once again had a living body they could trust. However, some did experience that, despite feeling stronger, they were beset by melancholy and fatigue after the operation.

This shouldn't be a problem any more… now this thing is out of the way, so to speak.

I feel lethargic and a bit off still, and then what that's down to I don't know. And the strange thing is that I, and we did read that you could have a bit of a dip psychologically and that, and I think it's strange because I mean, the operation has been done so, and it went well and you wake up after the operation and it's okay… so it shouldn't be a bother but strangely enough, it can be and I sit here and wonder, well, how was that now?

### 4.3 Interpretation and understanding (Step 3)

**FIGURE 3:**
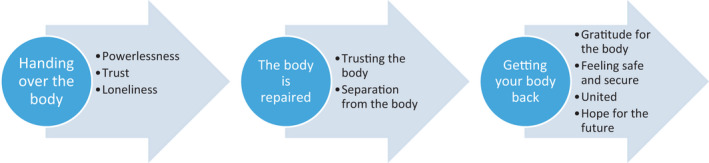
A transition of feelings and experiences of the body in relation to disease and treatment

**FIGURE 4:**
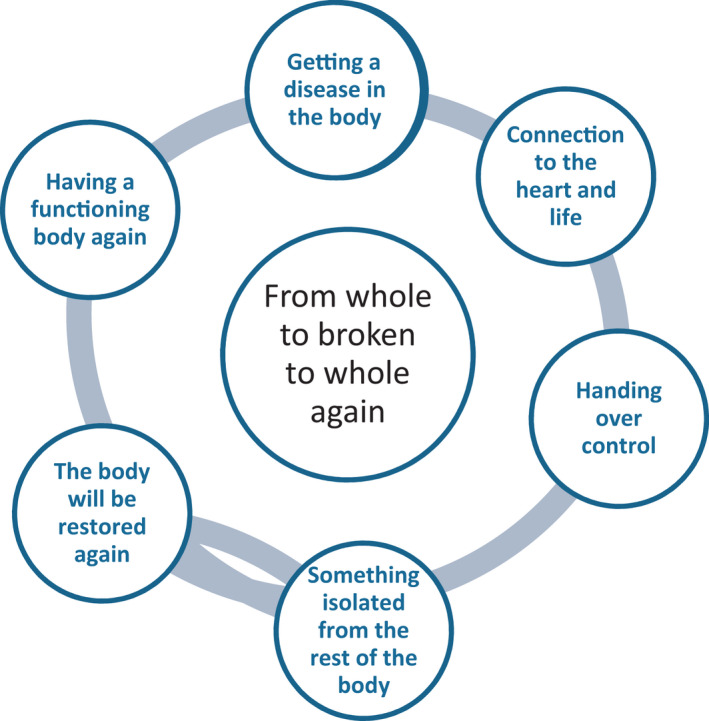
A summary of the understanding of the transition process that patients undergo (Main theme and themes from all interpretation steps (steps 1,2,3)

Patients’ relationship to their body and bodily function in connection with open surgery of AAA can be understood as a movement in three phases – a transition from one condition to another (Figure 3). The first transition is the movement from being healthy to the necessity to hand over the body to the surgeon and the healthcare personnel, the second is to have the body repaired and finally to get the body back again, Figure 4. These transitions can be understood as experiences of being broken or out of order, then repaired and whole again. Thus, the awareness of the body and its function becomes in focus, compared to the usual condition when we expect that the body does not bother us. In line with Gadamer’s (1996 Enigma of Health) thoughts, health manifests itself in a common feeling of well‐being and that there is a movement towards wholeness and equilibrium and thus health. Moreover, Gadamer (1996) has the opinion that it is not possible to treat the body without taking the whole of being into consideration.

## 5 DISCUSSION

The main findings of this study were that the patients went through a process or transition from being healthy to be broken and then to feeling whole again. In the theory of transition, Meleis (2010) describes the transition as a process of slow changes over time, where patients in a transition process are more vulnerable. In the interviews, the patients said this vulnerability both directly and indirectly. It can be understood, as a journey where the body forces the patient to realize the fragility of life and not only the image of their body.

The patients felt that they had no choice because the AAA threatened their life and their effort was to regain a functioning body and a “normal” life again, thus a movement towards health and well‐being (Gadamer, 1996).

The first transition consists of discovering the aneurysm in the body and realizing surgery is imminent – becoming a patient, someone suffering, even though this suffering was bearable, but also an obvious awareness of the body and life. It was evident that some patients realized the gravity of their situations and receiving the diagnosis felt life‐threatening. This situation brought with it a sense of powerlessness in having been afflicted by an insidious pathological and life‐threatening change in the body but also powerlessness in not having been able to do anything about this situation themselves. This concurs with previous research that shows the disease as being perceived as life‐threatening and that the body did not give any signal that the disease was present (Hansson et al., 2012; Pettersson & Bergbom, 2010).

In the second transition, the participants experienced handing over their bodies and along with it, responsibility for body and life to the surgeon and the nurses, which they trusted. This could be seen as both an active and passive act, in simply allowing others to do what has to be done. The patients did not really feel they had a choice because they were dependent on having the surgery if they wanted to live or avoid a threat to their lives. As there was no real alternative to surgery in these situations, the patients were forced into vulnerability and dependence on others. Handing over to others means others not only have to take good care of the patient's body but also the patient as a human being, and the patient's life. It was also found that the intervention described in patients’ drawings could be understood as repairing or fixing the body parts that were “broken” and that the surgeon could fix this. The patients’ trust and confidence in the healthcare professionals’ willingness to care for them seem to be connected to their continuing relationships established during the procedure (Westerling & Bergbom, 2008).

It is also important that healthcare staff listen to the patients’ wishes to participate, to help them feel secure and reduce a sense of vulnerability and dependence. A previous study about participation and decision‐making shows that factors that affected patient participation included patients’ willingness, nurses’ approach and confusion around expectations and roles (Tobiano et al., 2015).

The third and final transition from handing over the body and life to others to receiving it back again and thus regaining wholeness can be interpreted and understood as a restoration of the body but also of personal autonomy, independence and a uniting of body and mind. In this restoration, the aneurysm and operation are something the patient can leave behind them and return to “normal” life without having a threat to life hanging over them. In this phase of the transition to the reunion, some patients experienced tiredness and limited mobility, leading to a downheartedness that they had not expected. Similar experiences were found and discussed by Letterstål et al. (2017). They found that if the patients do not understand the postoperative impact on daily life, they are unprepared for complications and limitations during the recovery period. Several studies have reported decreased mental and physical quality of life following OR (Akbulut et al., 2018; Pettersson et al., 2012). However, from a long‐term perspective, other life circumstances also affect patients’ recovery and well‐being (Pettersson & Bergbom 2018). Also (Bruin et al. 2009) reported that in longer‐term follow‐up after surgery, patients’ quality of life and health status improved.

After being diagnosed, several patients reported that their relatives were more worried than they were. Interestingly, Eriksson et al. (2019) also describe relatives’ experiences of living with someone and knowing the risks of rupture. In that study, the relatives stated that the diagnosis was constantly on their minds, which also affected and limited daily activities. This may indicate the importance of involving relatives in the care process as participants, which in turn may lead to some kind of security and confidence. Thus, generating dialogue about recovery with both patients and relatives pre‐ and postoperatively is needed to foster realistic expectations and understanding of patients’ after‐care. After‐care planning should be done in cooperation between patient and nurse so that the patients feel they are participating and have some influence (Dahlberg, 2019).

The body's recovery is described as important for regaining a sense of health and being whole, whereby the body becomes a partner in the recovery process. According to Merleau‐Ponty (1997), we do not have a body but we are in our body, which is in line with how the patients in this study describe their experiences in the first and last transition process. In the second it seems that patients hand over their bodies to the professionals. This can be understood either as patients still are their bodies, or have a body that they hand over. The intervention and graft are something alien to the patient's being the body, as occurring together with their bodies, meaning they are linked. Recovery and reunification may be understood as a movement toward wholeness and that body, soul and spirit are an entity.

The drawings showed the patients perceived the graft as a tube and understood it as something separate from the rest of the body, a kind of spare part not connected to the aorta as a vital function. Patients also had doubts as to whether the graft would hold up and wondered about its effect on their daily lives. In a study by Persson and Hellström (2002) there were similar findings, with patients experiencing their bodies as alienated after a stoma operation. From the patient drawings in our study, it was clear that patients found it difficult to comprehend the information about the placement of the graft. They said they were unsure of what and where the intervention was. Some patients’ drawings illustrated the close connection between the intervention and the heart, which is understood as the patients realizing the seriousness of their condition and situation. The connection to the heart and life could also be understood as a metaphor for life where the operation of the aorta was vital for life and existence.

### 5.1 Limitations

One limitation of the method may be that pre‐understanding could have influenced the results, as one of the authors has long experience caring for people who have undergone surgery for AAA. This could also be a strength, as the results may become more clinically relevant and reflect the patient's experience in this context, however, the researcher was not the nurse in charge of the participant in this study. The fact that the other authors did not read the interview transcripts while interpreting the drawings could also contribute to strength and credibility. Quoting from the patient interviews further contributes to the credibility of the results (Polit & Beck, 2017). Moreover, in this study, there was a clear relationship between what the patients draw and what they described in connection with the interviews.

## 6 CONCLUSION

The main findings of this study were that the patients went through a process or transition from being healthy to being broken and then feeling whole and restored again. Going from broken to the whole can be seen as a transition between different conditions or as a continuous movement between health, ill‐health and health. The results also highlight the importance of gaining knowledge of patients’ own experiences of their body and bodily function in connection with surgery for open abdominal surgery.

## ETHICAL APPROVAL

The Regional Ethical Review Board (diary no. 560‐14).

## CONFLICT OF INTEREST

No conflict of interest has been declared by the authors.

## Data Availability

The data that support the findings of this study are available on request from the corresponding author (MP). The data are not publicly available due to privacy or ethical restrictions.

## References

[nop21225-bib-0001] Akbulut, M. , Aksoy, E. , Kara, İ. , Cekmecelioglu, D. , & Koksal, C. (2018). Quality of life after open surgical versus endovascular repair of abdominal aortic aneurysms. Brazilian Journal of Cardiovascular Surgery, 33(3), 265–270. 10.21470/1678-9741-2017-0236 30043919 PMC6089137

[nop21225-bib-0002] Bergbom, I. , & Lepp, M. (2021). Visual arts and drawings to communicate and explore authentic life situations, a data collection method in caring science ‐ a hermeneutic perspective. Scandinavian Journal of Caring Science, 1–10. 10.1111/scs.13040 34779536

[nop21225-bib-0003] Bergbom, I. , Modh, A. C. , Lundgren, I. , & Lindwall, L. (2017). First time pregnancy women’s experiences of their body in early pregnancy. Scandinavian Journal of Caring Sciences, 31(3), 579–586. 27726170 10.1111/scs.12372

[nop21225-bib-0004] Bruin, J. , Groenwold, R. H. A. F. , Brownrigg, J. R. , Prinssen, M. , Grobbee, D. E. , & Blankensteijn, J. D. , DREAM Study Group . (2009). Quality of life from a randomized trial of open and endovascular repair for abdominal aortic aneurysm. British Journal of Surgery, 302, 1535‐1542. 10.1001/jama.2009.1426 27059152

[nop21225-bib-0005] Dahlberg, H. (2019). Beyond the absent body—A phenomenological contribution to the understanding of body awareness in health and illness. Nursing Philosophy, 20(2), e12235 30773775 10.1111/nup.12235

[nop21225-bib-0006] Eriksson, A. , Carlsson, E. , Siu‐Yin, C. S. , Molassiotis, S. , & Kumlien, C. (2019). Partners’ experiences of living with men who have screening‐detected abdominal aortic aneurysms: A qualitative descriptive study. Journal of Clinical Nursing, 29, 3711–3720. 10.1111/jocn.15399 32619284

[nop21225-bib-0007] Fleming, V. , Gaidys, U. , & Robb, Y. (2003). Hermeneutic research in nursing: Developing a Gadamerian‐ based research method. Nursing Inquiry, 10(2), 113–120. 10.1046/j.1440-1800.2003.00163.x 12755860

[nop21225-bib-0008] Furnham, A. , Badmin, N. , & Sneade, I. (2002). Body image dissatisfaction: Gender differences in eating attitudes, self‐esteem, and reasons for excercise. The Journal of Psychology Interdisciplinary and Applied, 136(6), 581–596. 12523447 10.1080/00223980209604820

[nop21225-bib-0009] Gadamer, H.‐G. (1989). Truth and method. Continuum.

[nop21225-bib-0010] Gadamer, H.‐G. (1996). The enigma of health. The art of healing in a scientific age. (J. Gaiger & N. Walker, Trans.). Stanford University Press.

[nop21225-bib-0011] Guillemin, M. (2004). Understanding illness: Using drawings as a research method. Qualitative Health Research, 4(2), 272–289. 10.1177/0308022614562791 14768462

[nop21225-bib-0012] Hansson, A. , Brodersen, J. , Reventlow, S. , & Pettersson, M. (2012). Opening Pandora's box: The experiences of having an asymptomatic aortic aneurysm under surveillance. Health Risk & Society, 14(4), 341–359. 10.1080/13698575.2012.680953

[nop21225-bib-0013] Högberg, Å. (1996). Att utvecklas med symboler [To grow with symbols]. Natur och Kultur.

[nop21225-bib-0014] Lederle, F. A. , Wilson, S. E. , Johnson, G. R. , Reinke, D. B. , Littooy, F. N. , Acher, C. W. , Ballard, D. J. , Messina, L. M. , Gordon, I. L. , Chute, E. P. , Krupski, W. C. , Busuttil, S. J. , Barone, G. W. , Sparks, S. , Graham, L. M. , Rapp, J. H. , Makaroun, M. S. , Moneta, G. L. , Cambria, R. A. , … Bandyk, D. (2002). Immediate repair compared with surveillance of small abdominal aortic aneurysms. The New England Journal of Medicine, 346(19), 1437–1444. 10.1056/NEJMoa012573 12000813

[nop21225-bib-0015] Letterstål, A. , Eldh, A. C. , Olofsson, P. , & Forsberg, C. (2017). Patients’ experience of open repair of abdominal aortic aneurysm‐‐preoperative information, hospital care and recovery. Scandinavian Journal of Care Science, 31(3), 579‐586. 10.1111/scs.12372 21040016

[nop21225-bib-0016] Lindwall, L. (2004). Kroppen som bärare av hälsa och lidande. Åbo Akademis förlag.

[nop21225-bib-0017] Lindwall, L. , & Bergbom, I. (2009). The altered body after breast cancer surgery. International Journal of Qualitative Studies on Health and Well‐being, 4, 280–287. 10.3109/17482620903106645

[nop21225-bib-0018] Malina, M. , Nilsson, M. , Brunkwall, J. , Ivancev, K. , Resch, T. , & Lindblad, B. (2000). Quality of Life before and after endovascular and open repair of asymptomatic AAAs: A prospective study. Journal of Endovascular Therapy, 7(5), 372–379. 10.1177/152660280000700504 11032255

[nop21225-bib-0019] Meleis, A.‐I. (2010). Theoretical development of transitions. I A‐I. Meleis (Red.), *Transitions theory* (s. 13‐51). Springer Publishing Company.

[nop21225-bib-0020] Merleau‐Ponty . (1997). Kroppens fenemenologi (phenomenology of the body). Göteborg.

[nop21225-bib-0021] Modh, C. , Lundgren, I. , & Bergbom, I. (2011). First time pregnant women’s experiences in early pregnancy. International Journal of Qualitative Studies in Health and Well‐being, 6, 1–11. 10.3402/qhw.v6i2.5600 PMC3077216 21499449

[nop21225-bib-0022] Persson, E. , & Hellström, A. L. (2002). Experiences of Swedish men and women 6 to 12 weeks after ostomy surgery. Journal of Wound Ostomy Continence Nursing, 29(2), 103–108. 10.1067/mjw.2002.122053 11901419

[nop21225-bib-0023] Pettersson, M. , & Bergbom, I. (2010). The drama of being diagnosed with an aortic aneurysm and undergoing surgery for two different procedures: Open repair and endovascular techniques. Journal of Vascular Nursing, 28(1), 2–10. 10.1016/j.jvn.2009.10.001 20185074

[nop21225-bib-0024] Pettersson, M. , Bergbom, I. , & Mattsson, E. (2012). Health related quality of life after treatment of abdominal aortic aneurysm with open and endovascular techniques ‐ a two year follow up. Surgical Sciences, 9(3), 436‐444. Impact Factor: 0.3

[nop21225-bib-0025] Pettersson, M. , & Bergbom, I. (2018). Life is about so much more: Patients’ experiences of health, well‐being, and recovery after operation of abdominal aortic aneurysm with open and endovascular treatment‐A prospective study. Journal of Vascular Nursing, 37(3), 160–168. 10.1016/j.jvn.2019.06.002 31727308

[nop21225-bib-0026] Phillips, J. , Ogden, J. , & Copland, C. (2015). Using drawings of pain‐related images to understand the experience of chronic pain: A qualitative study. British Journal of Occupational Therapy, 78(7), 404–411. 10.1177/0308022614562791

[nop21225-bib-0027] Polit, D. F. , & Beck, C. T. (2017). Nursing research: Generating and assessing evidence for nursing practice (9th ed.). Lippincott.

[nop21225-bib-0028] Price, B. (1990). A model for body‐image care. Journal of Advance Nursing, 5(15), 585–593. 10.1111/j.1365-2648.1990.tb01858 2358576

[nop21225-bib-0029] Reimerink, J. J. , van der Laan, M. J. , Koelemay, M. J. , Balm, R. , & Legemate, D. A. (2014). Systematic review and meta‐analysis of population‐based mortality from ruptured abdominal aortic aneurysm. British Journal of Surgery, 101(7), 794–801, 9. 24037558 10.1002/bjs.9235

[nop21225-bib-0030] Rose, G. (2007). Visual Methodologies: An introduction to the interpretation of Visual Materials. Sage.

[nop21225-bib-0031] Sonesson, G. (1989). Pictorial concepts. Lund University Press.

[nop21225-bib-0032] Swedvasc . (2011). Nationella kvalitetsregistret för kärlkirurgi (Vascular surgery register). Verksamhetsrapport.

[nop21225-bib-0033] Swedvasc . (2020). Nationella kvalitetsregistret för kärlkirurgi (Vascular surgery register). Verksamhetsrapport.

[nop21225-bib-0034] Thorne, S. (2020). Beyond theming: Making qualitative studies matter. Nursing Inquiry, 27(1), 1–2. 10.1111/nin.12343 31990117

[nop21225-bib-0035] Tobiano, G. , Marshall, A. , Bucknall, T. , & Chaboyer, W. (2015). Patient participation in nursing care on medical wards: An integrative review. International Journal of Nursing Studies, 52(6), 1107–1120. 10.1016/j.ijnurstu.2015.02.010 25769475

[nop21225-bib-0036] Van Manen, M. (1998). Modalities of body experience in illness and health. Qualitative Health Research, 8(1), 7–24. 10.1177/104973239800800102

[nop21225-bib-0037] Westerling, K. , & Bergbom, I. (2008). The importance of nursing in perioperative care: A patient’s perspective. Journal of Avancerad Perioperative Care, 3(4), 133–214.

